# Cognitive remediation in the era of new technologies: Applications and innovations

**DOI:** 10.1192/j.eurpsy.2021.149

**Published:** 2021-08-13

**Authors:** T. Wykes

**Affiliations:** Department Of Psychology, Institute of Psychiatry, Psychology and Neuroscience, King’s College London, London, United Kingdom

**Keywords:** cognitive therapy, schizophrénia, cognitive remediation, psychologicaL therapy

## Abstract

Most cognitive remediation therapies now involve computer presentation that differ in their level of sophistication and incorporation of gaming technology. But sophistication doesn’t seem to affect the benefits as few outcome differences have been noted. Rather there seems to be a need for some interaction between a therapist and client with two recent meta-analyses reporting this therapist effect. For the large-scale roll-out of cognitive remediation this poses a problem – how do we train these therapists? We know that training or at least educational background is important, so we need clear training packages and supervision. Covid-19 has also given us a greater challenge as it has limited our face-to-face interactions. To remove these two challenges we can use technology. For training we need online processes to increase training availability and for a lack of face to face contact we can provide the bridge with suitable platforms which allow the sharing of screens. Both would ensure that cognitive remediation is available to a wider group, although that requires overcoming the digital divide often experienced by people with a diagnosis of schizophrenia. The tools and the training programme issues are discussed with reference to some initial data.

**Disclosure:**

No significant relationships.

